# Analysis of Fatigue Crack Paths in Cold Drawn Pearlitic Steel

**DOI:** 10.3390/ma8115388

**Published:** 2015-11-04

**Authors:** Jesús Toribio, Beatriz González, Juan-Carlos Matos

**Affiliations:** Fracture and Structural Integrity Research Group, University of Salamanca, E.P.S., Campus Viriato, Avda. Requejo 33, 49022 Zamora, Spain; bgonzalez@usal.es (B.G.); jcmatos@usal.es (J.-C.M.)

**Keywords:** pearlite, cold drawing, fatigue crack path, micro-roughness

## Abstract

In this paper, a fracto-metallographic analysis was performed on the cracked specimens of cold drawn pearlitic steel subjected to fatigue tests. Fatigue cracks are transcollonial and exhibit a preference for fracturing pearlitic lamellae, with non-uniform crack opening displacement values, micro-discontinuities, branchings, bifurcations and frequent local deflections that create microstructural roughness. At the micro-level, the cold drawn pearlitic steel exhibits higher micro-roughness than the hot rolled bar (this is a consequence of the manufacturing process by cold drawing), so that the actual fractured surface in the cold drawn wire is greater than that in the hot rolled bar, due to the fact that the crack deflection events are more frequent and with higher angle in the former (the heavily drawn prestressing steel wire). These findings show the relevant role on the manufacturing process by cold drawing in the fatigue crack propagation in pearlitic steel.

## 1. Introduction

Two-parameter approaches are very useful to analyze fatigue crack growth in engineering materials [1,2,3]. In this case, two driving forces are usually considered as parameters governing the evolution of the crack subjected to cyclic loading, e.g., Δ*K* and *K*_max_, or Δ*K* and *R*. Research carried out by Kujawski [4] shows that, in the case of ductile materials, the crack driving force for fatigue is dominated by the Δ*K* parameter whereas in the case of brittle materials it is governed by *K*_max_. The concept of an effective stress intensity factor (SIF) range Δ*K*_eff_ is discussed in [5], assuming the idea of fatigue crack closure proposed by Elber [6].

The crack path developed by fatigue (cyclic) loading is influenced by microstructural features of the material. In the case of ferritic-pearlitic steels, the crack advances along the ferritic seam through the grain boundaries [7]. In steel with pearlite uniformly distributed in ferrite, the fatigue cracking path is more tortuous than in those with isolated distribution, with larger angle deflections appearing during the crack advance [8]. In eutectoid steel with fully pearlitic microstructure, the crack tends to break the ferrite/cementite lamellae. In this case, the kind of fatigue fracture surface can be classified as transcollonial fracture [9].

In banded ferritic-pearlitic steels, the bands of pearlite (oriented in preferential directions) diminish the fatigue crack propagation rate, since they produce a more tortuous crack path, with more frequent and more angled deflections and branchings [10]. The tortuous fatigue crack path frequently produces a crack interlock and the crack branching reduces the local crack tip driving forces for its propagation [10]. The orientation of ferrite/cementite lamellae in fully pearlitic steels produces a retardation in the fatigue crack growth rate [9,11], because the cementite lamellae behave as serious obstacles for dislocation movement and therefore for crack propagation. Moreover, an increase in roughness of the fatigue fracture surface can also be detected [9]. In the framework of the fracture mechanics approach, the non-linear crack configuration can be taken into account [12]. In addition, variations in crack deflection features influence considerably the fatigue crack propagation rates and threshold SIF range values [13,14].

## 2. Experimental Method

A progressively drawn pearlitic steel (eutectoid chemical composition: 0.789% C, 0.698% Mn, 0.226% Si, 0.078% V, 0.071% Cr, 0.011% P, 0.005% S, 0.003% Al, balanced with Fe) was used in this work: from the hot rolled bar (not cold drawn at all) to the prestressing steel wire (obtained after seven cold drawing steps and a stress-relieving treatment), as well as the intermediate steps.

The stress-strain curves and the conventional mechanical properties were obtained by means of a standard tension test. The cold drawing process (up to cumulative plastic strain ε^P^ = 1.57) does not modify the Young’s modulus *E* (~200 GPa), and produces a clear improvement of material strength in the form of increase of both yield strength σ_Y_, from 700 MPa in the hot rolled bar to 1480 MPa in the cold drawn wire, and ultimate tensile strength (UTS) σ_R_, from 1220 MPa in the hot rolled bar to 1820 MPa in the cold drawn wire. However, the strain at UTS diminishes from 0.08 in the hot rolled bar to 0.06 in the cold drawn wire.

The fatigue tests were performed by applying a cyclic tensile load on cylindrical specimens taken from the bar and the wires (as received, 11.0 mm for the hot rolled bar and 5.1 mm for the cold drawn wire), and the crack growth was evaluated by means of compliance measurements. A sinusoidal wave was used with a frequency of 10 Hz and *R*-ratio ≈ 0. The maximum stress applied during the tests was always lower than the yield strength of the material. The crack front was characterized as a part of an ellipse with its center in the wire (cylinder) surface. The expression used for the calculation of the SIF *K* was that provided by Astiz [15] obtained the energy release rate under plane strain conditions using also the stiffness derivative technique on the basis of a virtual crack extension.

The fatigue fracture surfaces and the longitudinal cuts on the crack specimens, after its metallographic preparation and being etched with 4% Nital (mixture of 4 mL of nitric acid with 96 mL of ethanol) to reveal microstructure, they were examined by scanning electron microscopy, SEM. In all pictures, the crack propagation occurred from left to right.

## 3. Experimental Results

### 3.1. Microstructural Analysis

**Figure 1:**
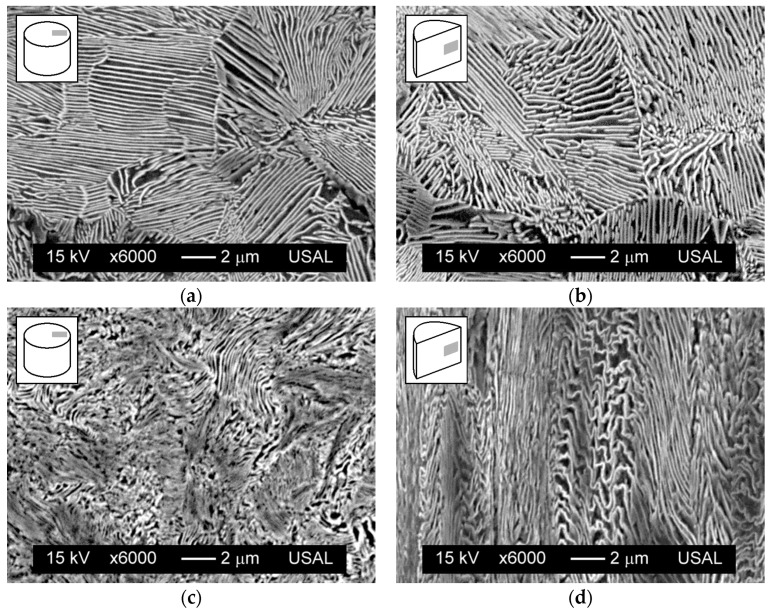
Microstructure of steel: hot rolled bar in transversal (**a**) and longitudinal (**b**) sections; cold drawn wire in transversal (**c**) and longitudinal (**d**) sections.

Figure 1 shows the microstructure of both materials (hot rolled bar and cold drawn wire), in their respective transverse and longitudinal sections. The horizontal axis of the micrograph corresponds to the radial direction in the wire, while the vertical axis of the micrograph is linked with the annular curvilinear coordinate in the transverse section of the wire and associated with the axial curvilinear coordinate in the longitudinal section of the wire.

The drawing process produces important microstructural changes in the steel at the two basic microstructural levels of pearlitic colonies and lamellae. The colonies become progressively enlarged (slenderized with change in aspect ratio) and oriented in axial direction (wire axis) with cold drawing. With regard to the lamellae, they are also axially oriented after drawing while at the same time the pearlite interlamellar spacing decreases with the level of cumulative plastic strain, thereby increasing the packing closeness. Therefore, the microstructure becomes progressively more dense and oriented with cold drawing.

### 3.2. Fractographic Analysis

**Figure 2:**
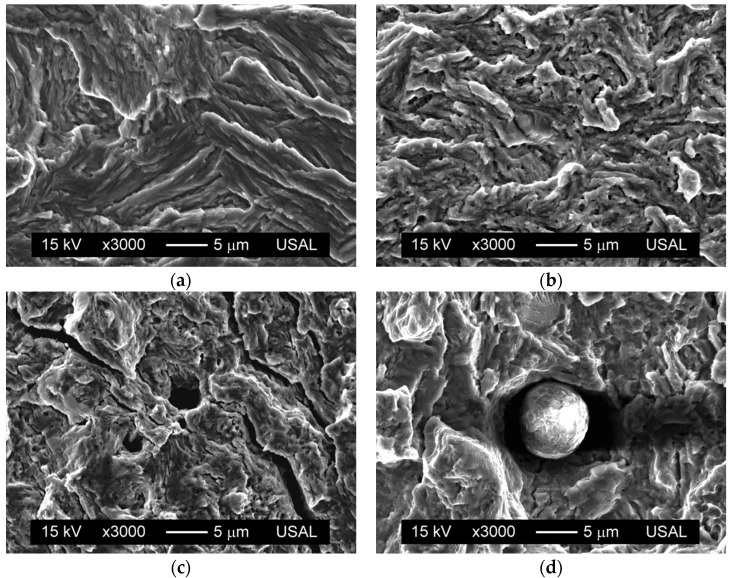
Fatigue fracture surface: (**a**) hot rolled bar, Δ*K* = 23 MPam^1/2^; (**b**) cold drawn wire, Δ*K* = 27 MPam^1/2^; (**c**) micro-cracking, Δ*K* = 42 MPam^1/2^; (**d**) particle, Δ*K* = 34 MPam^1/2^.

In eutectoid steels in which the microstructure is pearlitic, subcritical propagation by fatigue shows fracture in global mode I, so that the crack runs macroscopically along the wire’s cross section. At the microscopic level, the fatigue fracture surface exhibits ductile micro-tearing patterns (Figure 2) corresponding to highly-localized plastic strains, with no evidence of striation (characteristic of ductile metals and alloys).

In the case of heavily drawn prestressing steel, the ductile micro-tears are of smaller size and with curvier geometry than in the hot rolled wire (not cold drawn at all), as shown in Figure 2a,b, due to the microstructural changes (mostly in the wire’s cross section) produced by the high plastic strain undergone by the heavily cold drawn steel. On the matter of the fatigue surface, micro-cracks with a perpendicular direction to the advance of the crack are also observed (Figure 2c), whose presence is more frequent as either the SIF range Δ*K*, the *R*-ratio, or both of them increase. The existence of inclusions (sulphides, oxides, silicates, *etc.*) is also detectable in same cases on the fatigue surface (Figure 2d), although in many occasions only their trail is visible in the fractograph.

**Figure 3:**
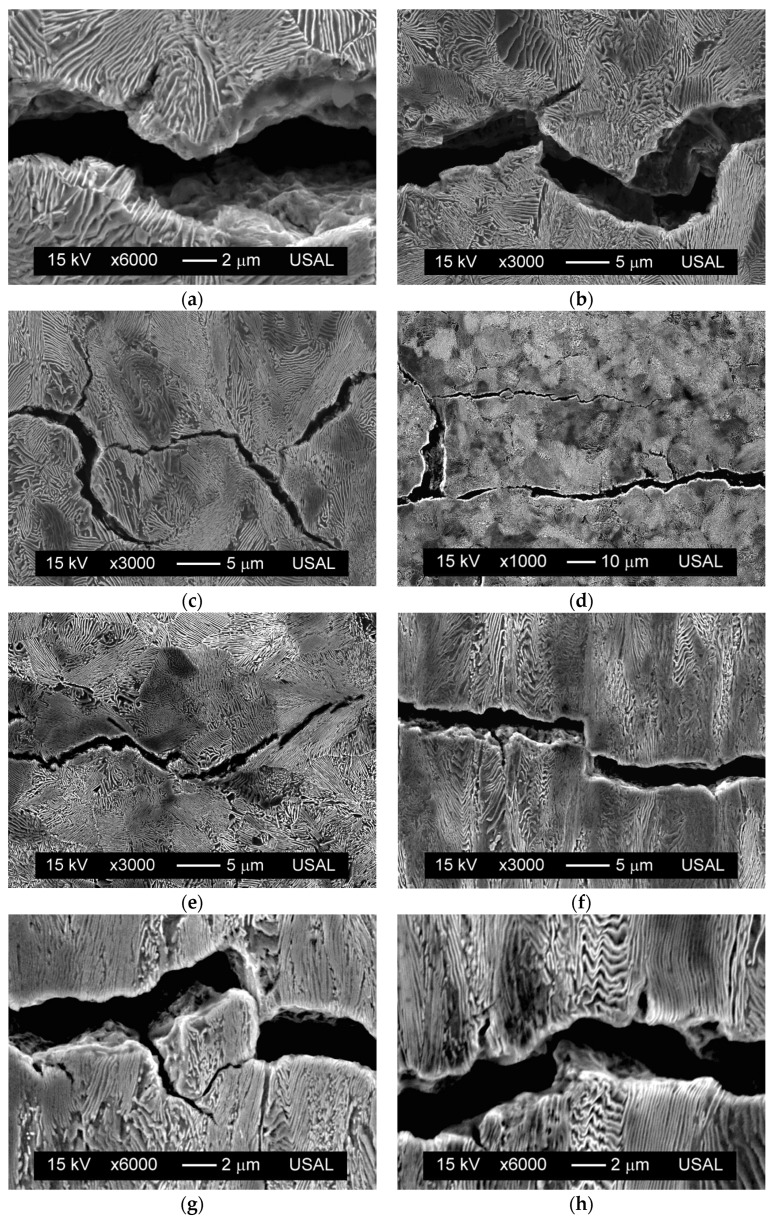
Crack paths, Δ*K* = 25 ÷ 40 MPam^1/2^: (**a**) micro-damage; (**b**) deflection; (**c**) branching; (**d**) multi-cracking; (**e**) micro-discontinuity; (**f**) interlocking; (**g**) debris; (**h**) pearlite pseudo-colony.

Figure 3 shows various longitudinal cuts of the crack path caused by fatigue in the steels studied. This crack is essentially transcollonial and translamellar, thereby tending to cross the pearlite colonies and to break the ferrite/cementite lamellae, showing highly localized plastic damage (Figure 3a). The crack propagation is tortuous, with frequent deflections (Figure 3b) or changes in the direction of crack advance and evidence of branching (Figure 3c), mainly bifurcations. In addition, the phenomenon of multicracking also appears sometimes (see Figure 3d). All of these events determine the existence, in the very-local close vicinity of the crack tip, of a mixed-mode fracture, so that fatigue cracking in this steel may be considered as locally multiaxial.

Sometimes, crack deflection appears next to a bifurcation, its appearance being more angular in this case. The crack branching shows the directionality of the fatigue advance, and is often in the form of branches on both sides of the macroscopic propagation plane, with an approximate angle between 45° and 90°, so that only one of the branches continues to grow and the other stops. These branches and bifurcations, observed in the longitudinal sections of the crack, correspond to the micro-cracks that appear on the metal surface fractured by fatigue (Figure 2c). These phenomena, crack deflection and bifurcation, cause surface micro-roughness and decrease the driving force for fatigue, thereby slowing the fatigue crack advance [8,10]. A retardation effect was experimentally observed in the fatigue propagation curves in the Paris region, whose fitting to the Paris-Erdogan law yields the same Paris exponent *m* for both steels (*m* ~ 3), while the constant *C* decreases from 5.3 × 10^−12^ MPa^−3^·m^−1/2^·cycle^−1^ for the hot rolled bar to 4.1 × 10^−12^ MPa^−3^·m^−1/2^·cycle^−1^ for the prestressing steel wire.

The fatigue cracks show continuous variations in the crack opening displacement COD, although this opening generally decreases from the crack mouth (origin of crack initiation) to the crack tip (final crack front). Moreover, in some regions the crack shows local micro-discontinuities during its propagation (Figure 3e). At the microscopic level, in the mixed mode crack advance (mode I + mode II), there are sections in which interlocking is observed [16], which corresponds to crack growth in the axial direction (mode II), resulting in a very small COD, which can even end up touching the fatigue fracture surfaces in a localized manner (Figure 3f).

The existence of *debris* (small loose particles of material on the fracture surface arising from the fatigue phenomenon [17], which become detached during the test) was not found, so that this phenomenon, if it takes place, occurs sporadically (Figure 3g). The presence of pearlite pseudo-colonies in strongly cold drawn steel (colonies whose pearlite lamellae, oriented in a nearly perpendicular direction in relation to the direction of drawing, having an anomalously high interlamellar spacing and which are wavy and sometimes broken) does not produce fatigue crack deflection (Figure 3h), unlike that which occurs in the fracture of those steels subjected to a greater number of drawing steps [18].

## 4. Discussion

**Figure 4:**
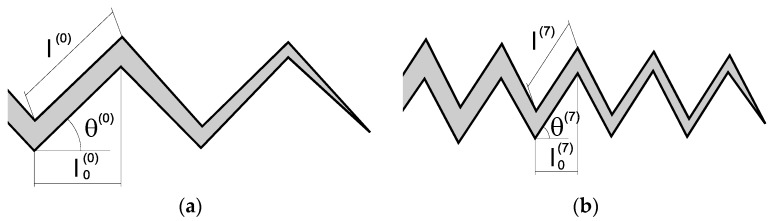
Fatigue crack paths in both materials: (**a**) scheme of the fatigue crack for hot rolled bar; (**b**) scheme of the fatigue crack for cold drawn wire.

The morphology of the crack path during fatigue was analyzed for both the hot rolled bar and the prestressing steel wire. The cracks produced by fatigue (cyclic) loading can be experimentally evaluated through the average deflection angle (θ) and the average deflection length (*l*) in the tortuous crack path, thereby detecting an evolution of parameters as a consequence of the strain hardening (and associated microstructural evolution) produced by the manufacturing process (cold drawing), as shown in the two parts of Figure 4. The superscripts (0) and (7) refer respectively to the hot rolled bar and the cold drawn wire, and the subscript 0 indicates that the length is projected over the main macroscopic crack advance direction (transverse to the wire axis).

As the degree of cold drawing increases, the average deflection angle rises θ^(7)^ > θ^(0)^. This experimental fact implies that the net fracture surface created by fatigue is higher in the drawn material than in the previous original steel (hot rolled bar). With regard to the average deflection length, it diminishes with cold drawing *l*^(7)^ < *l*^(0)^. This new experimental fact means that the deflections encountered in the fatigue fracture path are more frequent in the case of the commercial prestressing steel wire than in the base material before cold drawing (hot rolled bar).

Both phenomena of higher angle and more frequent (and shorter) deflections are probably linked with the microstructural evolution in the steel as a consequence of manufacturing by cold drawing, consisting of progressive orientation and slenderising of the pearlitic colonies, together with also a progressive orientation of ferrite/cementite lamellae and decreased of pearlitic interlamellar spacing (thus increasing the packing closeness). As a fracture mechanics effect of the previously described material science facts, a retardation of fatigue crack growth can be mentioned, thereby demonstrating that manufacturing of prestressing steel by cumulative wire drawing clearly improves the fatigue behavior of these steels. 

After analyzing different ranges of the applied SIF during fatigue crack propagation, the profiles followed by the fatigue crack in the longitudinal sections of the specimen could be evaluated. The profile length ratio λ (ratio of the actual length of the crack increment, *L*, to the length of its transverse projection, *L*_0_) was calculated:

(1) $$\lambda =\frac{L}{L_{0}}$$

**Figure 5:**
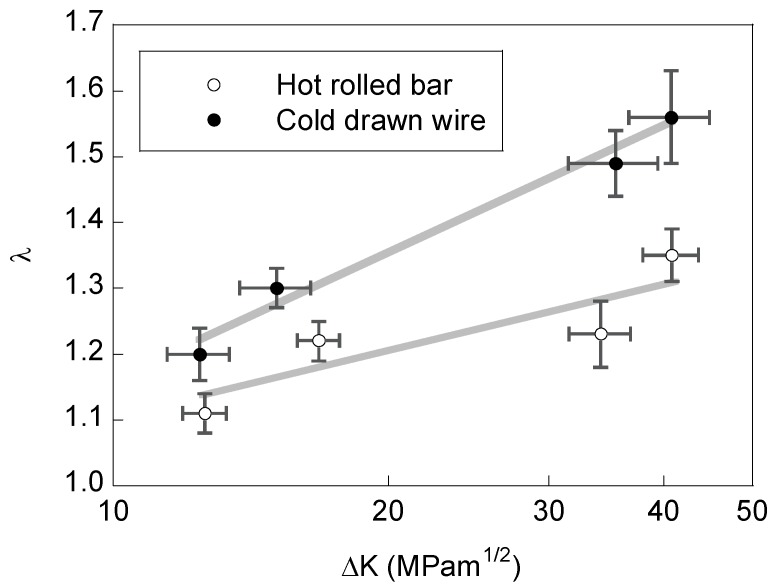
Profile length ratio, λ, *vs.* stress intensity factor range, Δ*K*, for the hot rolled bar and the cold drawn wire.

Experimental results (Figure 5) show that λ increases with both the cold drawing degree (as a consequence of the microstructural changes undergone by the steel during the manufacturing process by cold drawing) and the SIF range Δ*K* (as a consequence of plastic damage in the heavily stress area in the close vicinity of the crack tip). This parameter λ represents the asperity or roughness of the cracking path created by fatigue, *i.e.*, it involves both the angle of the locally deviated branches (deflection angle) and the appearance of secondary branches (cracking embryos) departing from the main crack path.

With regard to the two parameters governing the fatigue crack growth, both the SIF range Δ*K* and the *R*-ratio influence the features of the fracture surface created by fatigue (cyclic) loading. The increment of any of these parameters (the SIF range Δ*K* and the *R*-ratio) makes the typical micro-tearing features appearing in the fatigue surface more tortuous and pronounced [9].

At the meso level, the fatigue fracture surface of the base material (hot rolled bar, not cold drawn at all) develops with greater height variations than the prestressing steel (heavily drawn). However, at a finer micro-level, the latter exhibits higher micro-roughness, so that the actual fractured surface in the cold drawn wire is greater than that in the hot rolled bar.

With regard to the events related to crack deflections, branchings, bifurcations, *etc.*, they are more frequent and with higher angle in the prestressing steel wire than in the hot rolled bar. Therefore, the level of kinking and tortuosity of the crack increases, not only as it propagates and the SIF range Δ*K* increases, but also in the cold drawn wire when compared with the parent material (hot rolled bar, not cold drawn at all).

The afore-said level of kinking and tortuosity of the crack path is associated with an increase of the micro-crack deflection angle in the prestressing steel, the height of the deviation path in both materials (lower in the plastically strained steel), the number of deflections per projected length (more elevated in the cold drawn wire) and the general roughness of the fracture surface created by fatigue in both steels (hot rolled bar and prestressing steel wire).

## 5. Conclusions

The following conclusions have been drawn from this work regarding the fracto-metallographic analysis of fatigue cracking in cold drawn pearlitic steel:

(i) From the microscopic point of view, fatigue cracks in pearlitic steel develop exhibiting micro-plastic tearing. This fact is consistent with an evidence of cyclic micro-damage and crack advance produced by a mechanism of plastic strain concentration. The cold drawn wire exhibits a pattern resembling micro-tearing, these events being of lower size and more curved aspect than those associated with the hot rolled bar.
(ii) Cracking paths produced by fatigue (cyclic) loading develop in the form of trans-colonial advance and tending to fracture a certain proportion of pearlitic lamellae in the corresponding colony of pearlite. As a matter of fact, fatigue crack propagation can be classified as tortuous, with certain quantity of micro-discontinuities, branchings (frequently, bifurcations also appear), as well as local deflections, thereby producing a sort of roughness (at the microstructural level) with associated non-uniform crack opening displacement distribution.
(iii) The fractographic analysis of the cracked surface produced by fatigue fracture in the cold drawn pearlitic wire exhibits an appearance consisting of micro-roughness. In this case, the total fractured surface (including the afore-said micro-discontinuities, branchings, bifurcations and local deflections) is greater than in the case of the hot rolled bar (base material). The reason is that the deflections in the fatigue crack path are more frequent and with greater angle in the cold drawn wire than in the hot rolled bar. The increase of the stress intensity factor (SIF) range, ∆*K*, also produces higher micro-roughness in the fracture surface.
